# Improving production of *Streptomyces griseus* trypsin for enzymatic processing of insulin precursor

**DOI:** 10.1186/s12934-020-01338-9

**Published:** 2020-04-13

**Authors:** Yunfeng Zhang, Qixing Liang, Chuanzhi Zhang, Juan Zhang, Guocheng Du, Zhen Kang

**Affiliations:** 1 Key Laboratory of Industrial Biotechnology, Ministry of Education, Jiangnan University, 1800 Lihu Road, Wuxi, 214122 Jiangsu China; 2 The Key Laboratory of Carbohydrate Chemistry and Biotechnology, Ministry of Education, School of Biotechnology, Jiangnan University, Wuxi, 214122 China; 3 Center for Synthetic Biochemistry, Institute of Synthetic Biology, Shenzhen Institutes of Advanced Technologies, Shenzhen, China; 4 Bio-Pharmaceutical Research Institute Lian Yun Gang Chia Tai Tianqing Pharmaceutical Group Co., Ltd, Lianyungang, Jiangsu China

**Keywords:** *Streptomyces griseus* trypsin, Autolysis, Unfolded protein response (UPR), *Pichia pastoris*, Insulin

## Abstract

**Background:**

Trypsin has many applications in food and pharmaceutical manufacturing. Although commercial trypsin is usually extracted from porcine pancreas, this source carries the risks of infectivity and immunogenicity. Microbial *Streptomyces griseus* trypsin (SGT) is a prime alternative because it possesses efficient hydrolysis activity without such risks. However, the remarkable hydrolysis efficiency of SGT causes autolysis, and five autolysis sites, R21, R32, K122, R153, and R201, were identified from its autolysate.

**Results:**

The tbcf (K101A, R201V) mutant was screened by a directed selection approach for improved activity in flask culture (60.85 ± 3.42 U mL^−1^, increased 1.5-fold). From the molecular dynamics simulation, in the K101A/R201V mutant the distance between the catalytical residues D102 and H57 was shortened to 6.5 Å vs 7.0 Å in the wild type, which afforded the improved specific activity of 1527.96 ± 62.81 U mg^−1^. Furthermore, the production of trypsin was increased by 302.8% (689.47 ± 6.78 U mL^−1^) in a 3-L bioreactor, with co-overexpression of chaperones SSO2 and UBC1 in *Pichia pastoris*.

**Conclusions:**

SGT protein could be a good source of trypsin for insulin production. As a result of the hydrolysates analysis and direct selection, the activity of the tbcf (K101A, R201V) mutant increased 1.5-fold. Furthermore, the production of trypsin was improved threefold by overexpressing chaperone protein in *Pichia pastoris*. Future studies should investigate the application of SGT to insulin and pharmaceutical manufacturing.

## Background

Trypsin (EC 3.4.21.4) is an alkaline protease that has stringent cleavage specificity for the carboxyl termini of arginine (R) and lysine (K). Similar to other serine proteases, trypsin shares the canonical catalytic triad H57, D102, and S195 [1–3]. To date, trypsin has been widely used in leather bating, detergents, and the food and pharmaceutical industries. In particular, trypsin was also used in insulin manufacture to convert the insulin precursor into insulin ester by digesting the mini-C-peptide [4–7]. In food processing, trypsin was applied in the preparation of nutritional proteins/peptides [8], reducing the allergenicity and enhancing the digestibility of baby food [9]. Trypsin was also evaluated for its wound healing property in the pharmaceutical field [10]. Currently, commercial trypsin is usually extracted from porcine and bovine pancreas. However, due to the potential risk of contamination with infectious agents, animal-derived trypsin is under strict control in pharmaceutical and food manufacturing. Thus, microbial *Streptomyces griseus* trypsin (SGT) is a potential alternative that shares similarities with bovine trypsin (BT) in three-dimensional structure and catalytical activity. Moreover, SGT displays a higher hydrolysis rate than BT in proteomics applications because it produces an amount of matching tryptic peptides [11]. However, the high hydrolysis efficiency of SGT causes autolysis [12] because there are 16 potential autolysis residues in its polypeptide sequence. Thus, high yield production of trypsin might be hindered by the autolysis of active trypsin. It is known that the mutant tbcf (K101A), with R145I and R201V mutations, affords greater stability against autolysis [13]. However, the autolysis residues of SGT have not been investigated in detail compared with BT [14, 15].

Over the past decade, SGT has been studied in different microbial hosts, such as *Escherichia coli* [16], *Bacillus subtilis* [17], *S. griseus* [18, 19], and *Pichia pastoris* [20, 21]. Notably, by engineering the N-terminal peptide, Zhang et al. achieved high-yield production of trypsin (227.65 U mL^−1^) in *P. pastoris* [13, 22]. Recently, some studies have found that unfolding protein response and endoplasmic reticulum associated degradation happened in *P. pastoris* when expressing human trypsinogen protein [23, 24]. Thus, overexpression of chaperones might help to increase SGT production. In this study, our objective is to develop a strategy for the high-yield production of SGT in *P. pastoris* by rationally designed mutants of SGT and co-overexpression of chaperones to mitigate unfolded protein accumulation. Moreover, the SGT mutant could then be applied in manufacturing insulin from the insulin precursor.

## Results and discussion

### Identification of the autolysis sites in tbcf (K101A)

**Fig. 1:**
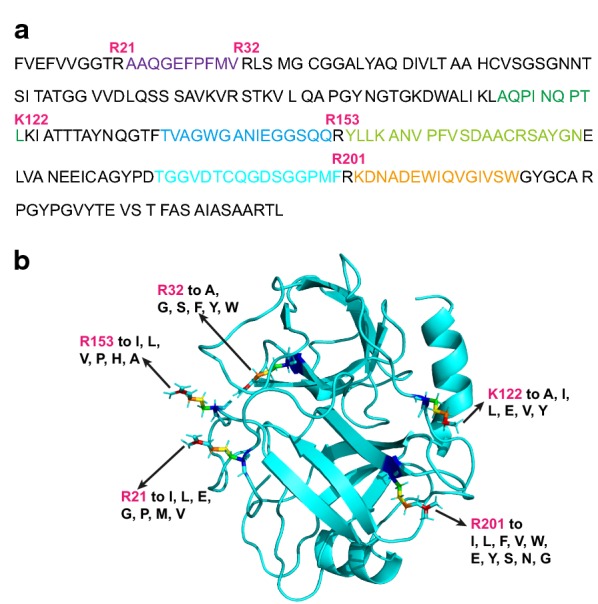
Identification of the autolysis residues and predicted mutations. **a** The peptide fragments detected from the hydrolysate of tbcf (K101A). The peptide fragments are presented in colored sequences. **b** The predicted mutations of autolysis residues in tbcf (K101A)

In last decade, the researches have engineered the regulator of *SGT* gene and optimized medium components to improve trypsin production in *Streptomyces* (Table 1). By engineering propeptide, Ling et al. successfully expressed the mature SGT sequence in *P*. *pastoris* and the activity reached 14.4 U mL^−1^ [20]. However, the autolysis of SGT hindered its higher production in *P. pastoris* [22]. SGT contains 16 potential autolysis residues (Arg, Lys). In the previous study, the stability and production of SGT were found to be improved in the tbcf (K101A) mutant [13]. To further investigate the autolysis of tbcf (K101A), its hydrolysate was analyzed by MALDI-TOF–MS. Based on the autolysis fragments of tbcf (K101A), five autolysis residues were identified: R21, R32, K122, R153, and R201 (Fig. 1a). This result indicated that SGT marginally preferred to hydrolyze at R rather than K, which may be because the hydrogen bond interaction between R and substrate-binding D189 is more stable than a water molecule bridged interaction between K and D189 [25]. Štosová et al. significantly reduced the autolysis of SGT by modifying R or K residues with the chemical reagents phenylglyoxal and formaldehyde, but the specific activity of SGT dropped to 12% of that of the parent enzyme [12]. In general, R and K interact with other residues to form hydrogen bonds, salt bridges, and π-interactions, and these interactions play essential roles in the folding, three-dimensional structure, and catalytic activity of SGT. In the secondary structure of SGT, except for R201V (β-Sheet), the other four autolysis residues (R21, R32, K122, and R153) were located in the loop regions (Fig. 1b). Specifically, R21 interacted with Y131 via a hydrogen bond, which was also the case for R32–T55 and R32–Q128 interactions. K122 forms a salt bridge with D184 and E188, as does R153 with D60. These results indicated that the R21, R32, K122, and R153 residues helped stabilize the three-dimensional conformation.

**Table 1:** The production of *Streptomyces* trypsin in different strains

Strains	Production	Strategies	References
*S. lividans* 1326	6.6 U mL^−1^	pWHM3- *sprT*, recombinant expression	[55]
*S. lividans* TK24	7.4 U mL^−1^	Recombinant expression	[56]
*S. griseus* IFO13350 (pWHM3-TR1R2)	12.84 U mL^−1^	Co-expressing positive regulatory genes *sgtR1*, *sgtR2*; medium optimization (5.5-fold increased)	[19]
*S. griseus* IFO13350 (pWHM3-TR1R2)	15.18 U mL^−1^	Expression vectors containing *sprT*, *sgtR1*, and *sgtR2*	[18]
*E. coli*	–	–	[16]
*Bacillus subtilis* SCK6	33.8 U mL^−1^	*Streptomyces* trypsin GM2938	[30]
*Pichia pastoris*	14.4 U mL^−1^	Mature SGT sequence; propeptide swap; fed-batch fermentation	[20]
*P. pastoris*	1.45 U mL^−1^	Propeptide engineering; 6.71-fold increased (flask culture)	[21]
*P. pastoris*	47.4 U mL^−1^	Auto-catalyzed N-terminal peptide; fed-batch fermentation	[22]
*P. pastoris* GS115-tbcf (101A)*	40.5 U mL^−1^	Pro-peptide mutation; artificial pro-peptide; single mutation of autolysis residues (flask culture)	[13]
*P. pastoris* GS115-tbcf (101A)	227.65 U mL^−1^	Fed-batch fermentation	[13]
GS115-tbcf (K101A, R201V)	60.85 U mL^−1^	tbcf (K101A, R201V) mutant, 1.5-fold increased (flask culture)	In study
GS115-tbcf (K101A, R201V)-UBC1	80.1 U mL^−1^	Overexpression UBC1, 1.98-fold increased (flask culture)	In study
GS115-tbcf (K101A, R201V)-SSO2	86.12 U mL^−1^	Overexpression SSO2, 2.13-fold increased (flask culture)	In study
GS115-tbcf (K101A, R201V)-SU	109.25 U mL^−1^	Overexpression SSO2 and UBC1, 2.7-fold increased (flask culture)	In study
GS115-tbcf (K101A, R201V)-SU	689.47 U mL^−1^	Fed-batch fermentation, threefold increased from GS115-tbcf (101A)	In study

–, Inclusion body

* The parent strain in this study

### Directed selection of SGT

**Fig. 2:**
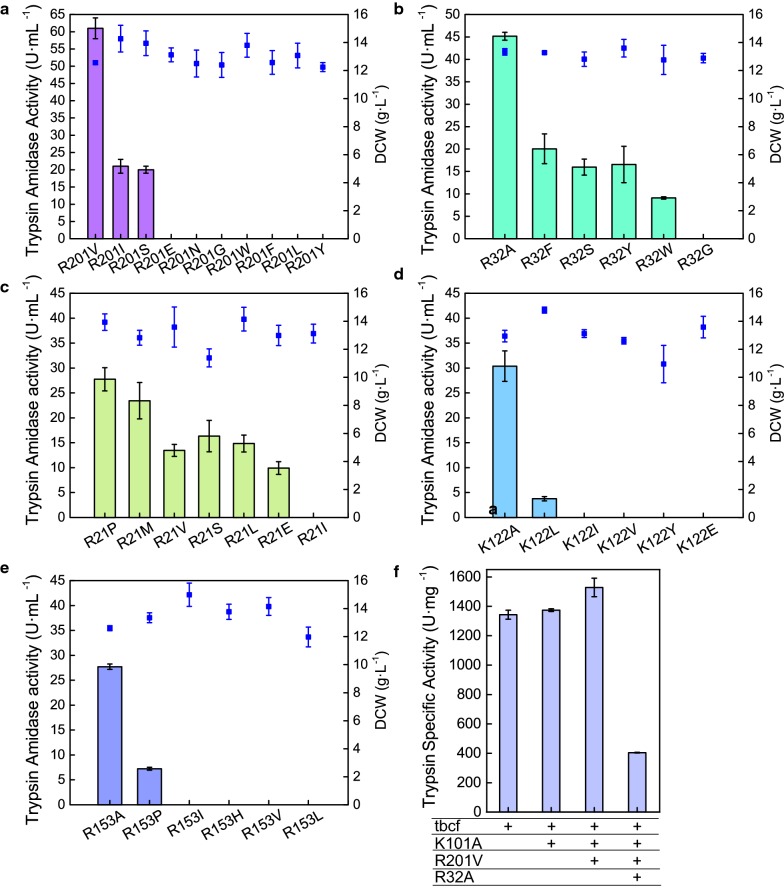
Amidase activity of the trypsin variants. Trypsin amidase activity (rectangle), DCW (filled square). **a** Variant of R201 mutation, **b** variant of R32 mutation, **c** variant of R21 mutation, **d** variant of K122 mutation, **e** variant of R153 mutation, and **f** the specific activity of the trypsin mutant

The direct selection method has been widely applied to engineer proteins with specific properties [26]. With the help of online bioinformatics tools, bespoke candidate mutations can be suggested according to the overall protein stability, residue physico-chemical environment, and interaction network of the protein [27–29]. SGT candidates were sorted for higher trypsin activity than the parent strain GS115-tbcf (K101A) in flask culture. At the C-terminal residue R201, the tbcf (K101A, R201V) mutant showed the highest activity of 60.85 ± 3.42 U mL^−1^, which was 1.5-fold greater than the activity of the parent strain [13] (Fig. 2a). Among six mutations of R32, R32A was outstanding with increased trypsin activity of 45.15 ± 0.99 U mL^−1^ (Fig. 2b). In contrast, other mutations of R21, K122, and R153 decreased the trypsin activity (Fig. 2c–e). The R32A mutation was then added to tbcf (K101A, R201V) to afford the tbcf (K101A, R201V, R32A) mutant. tbcf (K101A, R201V, R32A) displayed a higher expression level than tbcf (K101A, R201V) (Additional file 1: Fig. S1), although its activity decreased by 50% (30.67 ± 2.63 U mL^−1^). Further, the specific activity of the trypsin mutants was compared using the substrate BAPNA via a spectrophotometric assay. The mutant tbcf (K101A, R201V) showed the highest specific activity of 1527.96 ± 62.81 U mg^−1^, which was 13.76% greater than that of the parent. The results suggested that rational engineering of self-degradation sites is effective and this strategy should be also used for improving trypsin expression in prokaryotes [30].

### Enzyme kinetics and molecular modeling of the SGT mutant

**Fig. 3:**
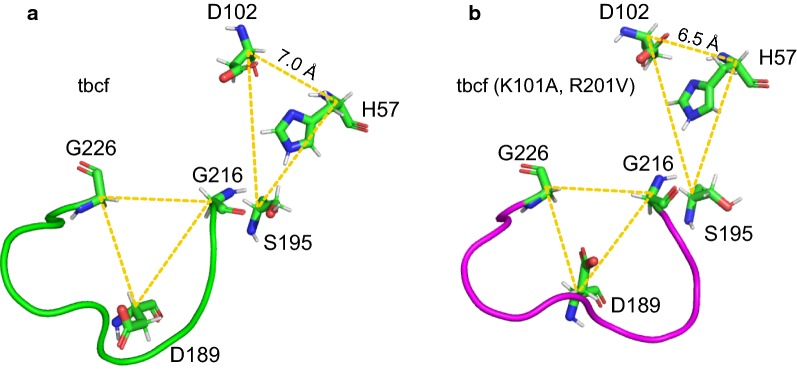
Comparison of the three-dimensional structures of the trypsin mutants tbcf (**a**) and tbcf (K101A, R201V) (**b**)

An MD simulation was applied to compare the three-dimensional structure of tbcf (K101A, R201V) with that of tbcf. The root-mean-square deviations (RMSD) of the protein backbone atoms gave an indication of the stability of the protein [31]. After the 7-ns MD simulation, the backbone of the tbcf (K101A, R201V) mutant exhibited a lower deviation of RMSD value (Additional file 1: Fig. S2). This result indicated that the K101A/R201V mutations could improve the stability of the SGT backbone. Then, the internal interactions of the catalytical triad (H57, D102 and S195) were analyzed. Interestingly, there was a shorter distance between H57 and D102 (6.5 Å vs 7.0 Å) in the catalytic center of the tbcf (K101A, R201V) mutant (Fig. 3a, b). Consequently, the tbcf (K101A, R201V) mutant, with a *k*_cat_/*K*_m_ value of 1.53 × 10^7^ min^−1^ mM^−1^, afforded higher catalytical activity than the parent tbcf (K101A) (Additional file 1: Table S3). This increased specific activity might be attributed to the shortened distance between D102 and H57 in the catalytical triad, which could consolidate the hydrogen bond between the carboxylic oxygen of D102 and δ-nitrogen of H57 [32]. Because this hydrogen bond would stabilize the structure of H57 in the catalytic transition state, this would facilitate the acceptance of a proton by H57 from S195 [33]. Moreover, the *K*_m_ values of tbcf (K101A, R201V) and tbcf were similar, at 5.39 ± 0.36 × 10^−2^ mM and 5.86 ± 0.16 × 10^−2^ mM, respectively. This result indicated that K101A/R201V mutations retained the conserved internal interactions at the substrate binding domain.

### High yield production of SGT with co-overexpression of chaperones in *P. pastoris*

**Fig. 4:**
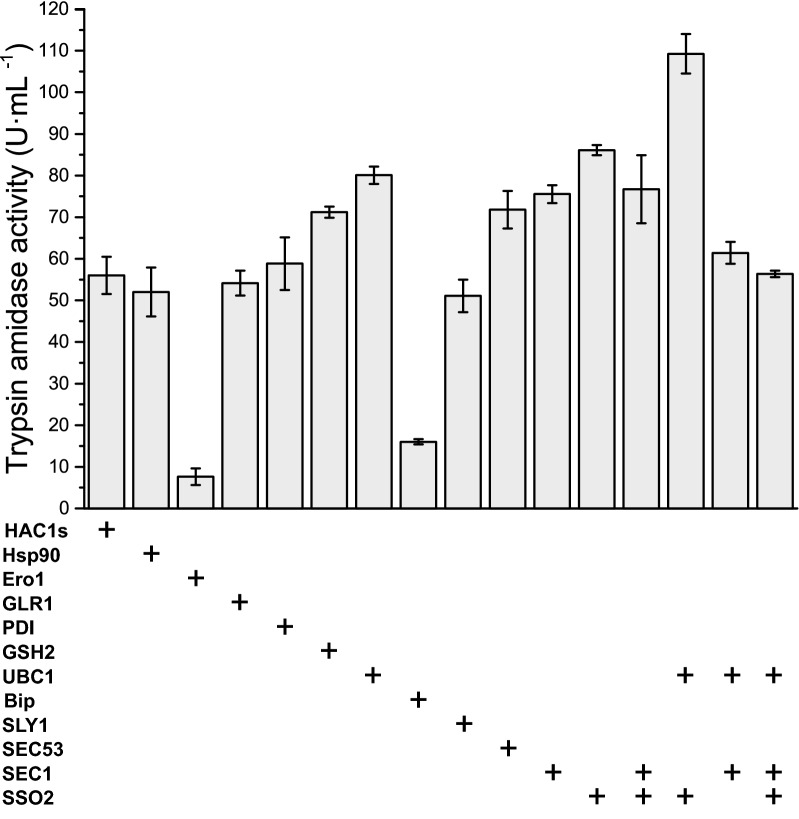
Trypsin amidase activity of the *Pichia pastoris* yeast strain with overexpression of the endogenous proteins

Protein expression is known to be regulated by UPR [34] or ERAD [35] in *P. pastoris*. Furthermore, the expression of trypsinogen triggers UPR and ERAD in *P. pastoris*, due to the presence of unfolded trypsinogen in the endoplasmic reticulum (ER), and peroxide toxicity, due to the formation of a disulfide bond [23, 24, 36]. It is known that protein expression can be improved by upregulating endogenous proteins [37]. Therefore, 12 proteins, which are involved in transcription regulation, disulfide bond formation, and protein secretion, were individually overexpressed. These ER-located chaperones conduct diverse functions during the process of polypeptide folding into the biologically active protein. Specifically, these chaperones are involved in the oxidative reaction that occurs during protein folding (Ero1), disulfide bond formation (GLR1, PDI, and GSH2), and degradation of the unfolded protein (UBC1) [35, 36]. Interestingly, the production of trypsin increased by 1.76-fold and 1.98-fold with overexpression of GSH2 and UBC1, respectively (Fig. 4). Moreover, the transport of polypeptides is known to be critical for secretory proteins. The Bip, SLY1, and SEC53 chaperones are responsible for transporting and recognizing nascent polypeptides in the ER and Golgi membrane [38]. SEC1 and SSO2 promote the extracellular secretion of the folded protein [39, 40]. So, in this case overexpression of SEC1 and SSO2 increased the trypsin activity by 1.86-fold and 2.13-fold, respectively. Then, SEC1, SSO2 and UBC1 were together co-overexpressed because they contributed more than a 20% increase in trypsin activity. Finally, the trypsin production of strain GS115-tbcf (K101A, R201V)_SU showed the highest production of 109.25 ± 4.76 U mL^−1^ (increased by 2.67-fold), with co-overexpression of SSO2 and UBC1 in flask culture (Fig. 4). This result indicated that the bottleneck that hinders high-yield production of SGT might be ameliorated by secretory transportation and degradation of unfolded trypsin in *P. pastoris*.

### Application of high yield trypsin to processing of the insulin precursor

**Fig. 5:**
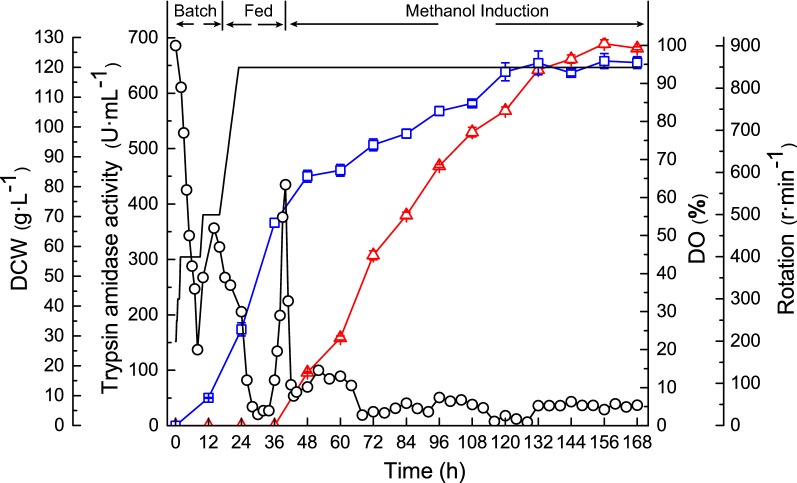
Fed-batch fermentation of the yeast strain in a 3-L bioreactor. Trypsin amidase activity (triangle), dry cell weight (DCW, square), dissolved oxygen (DO, circle), and rotation speed

To scale up the production of trypsin, the strain GS115-tbcf (K101A, R201V)_SU was cultured in a 3-L bioreactor, according to the published method [13]. After glycerol fed-batch cultivation, a higher density of cells (68.02 ± 1.5 g/L, DCW) was achieved with glycerol feeding and a high agitation speed (850 r min^−1^) (Fig. 5). Then, the fermentation entered the methanol-feeding cultivation phase, when the glycerol was depleted as indicated by increased DO (over 50%). After induction for 156 h, the trypsin production reached 689.47 ± 6.78 U mL^−1^, which was threefold higher than parent GS115-tbcf (K101A).

**Fig. 6:**
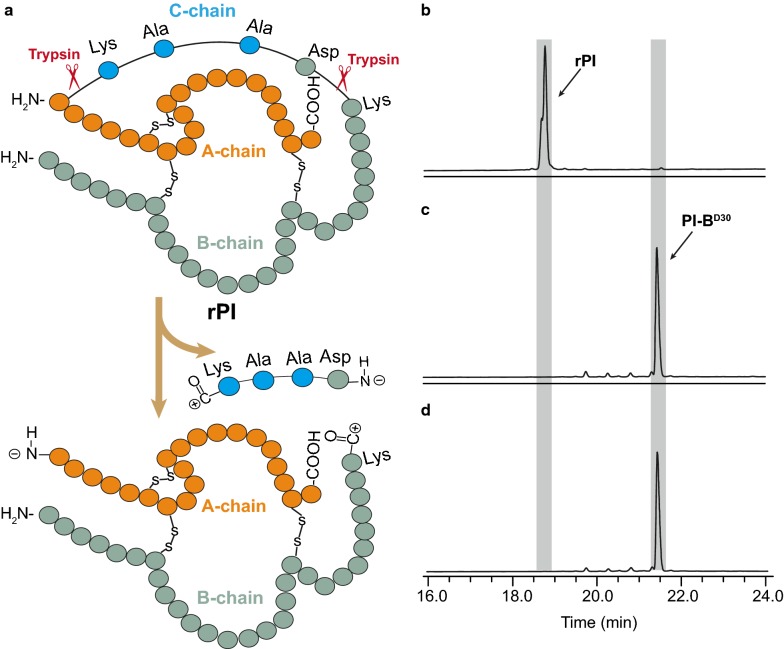
HPLC chromatograms of insulin manufactured with trypsin. **a** Schematic presentation of the hydrolysis process. rPI, recombination insulin precursor; PI-B^D30^, insulin precursor with Asp30-deleted B-chain. **b**–**d** Chromatograms of the hydrolysis products of rPI after incubation with trypsin at 25 °C for 19 h: **b** rPI, **c** rPI and commercial porcine trypsin, and **d** rPI and tbcf (K101A, R201V)

Mammalian trypsin is generally utilized for the preparation of insulin from its precursor because of the canonical tryptic cleavage of lysine to remove the C-chain [4, 41]. Most commercial trypsin is extracted from the mammalian pancreas, which carries the risks of bioactive compound contaminants, infectious viruses, and health-harming proteases [42]. Although the heterologous expression of mammalian trypsin could avoid the aforementioned problems, it still suffers from immunogenicity issues, a low expression level, activation of the zymogen, and autolysis [15, 43, 44]. Importantly, the SGT mutant tbcf (K101A, R201V) exhibited better hydrolysis performance with no immunogenicity, high production, autoactivation, and stability against autolysis. tbcf (K101A, R201V) was mixed with insulin precursor rPI to afford the insulin precursor with Asp30 deleted B-chain (PI-B^D30^) (Fig. 6a). And tbcf (K101A, R201V) was compared with commercial porcine trypsin under identical conditions. After hydrolysis for 19 h, rPI was converted to PI-B^D30^ as demonstrated by HPLC. The elution time of rPI was 18.75 min (Fig. 6b). After cleavage by commercial porcine trypsin and tbcf (K101A, R201V), rPI was converted to PI-B^D30^, which was eluted at 21.40 min (Fig. 6c, d). Hence, the engineered SGT mutant tbcf (K101A, R201V) performed the potential application of insulin manufacture with the same hydrolysis capacity as commercial porcine trypsin.

## Conclusions

In this study, five autolysis residues were identified in SGT. The mutant tbcf (K101A, R201V) was identified from a library of 35 mutants with improved hydrolysis performance and specific activity. Furthermore, the production of trypsin in *P. pastoris* was increased by 302.8% (689.47 ± 6.78 U mL^−1^) with co-overexpression of the chaperone proteins SSO2 and UBC1. Consequently, the engineered SGT tbcf (K101A, R201V) displayed the same hydrolysis capacity as commercial porcine trypsin. Future studies should investigate the application of SGT in the manufacturing of insulin and other pharmaceuticals [45].

## Methods

### Chemical and materials

PCR kit was purchased from Takara (Takara Ltd., Otsu, Japan). Restriction enzyme, 12% SDS-PAGE gel, Gel-extraction kit, and high-performance liquid chromatography (HPLC) column (Thermo scientific accucore C4 2.6 μm 50 × 4.6 mm 150A) were purchased from Thermo Scientific (Shanghai, China). DNA ligation kit (CloneExpress™ II) was purchased from Vazyme Biotech Co., Ltd. (Nanjing, China). *P. pastoris* GS115 (His^−^) and *E. coli* JM109 strain were stocked in the laboratory. Nα-benzoyl-dl-arginine-p-nitroanilide (BAPNA), Nα-benzoyl-l-arginine ethyl ester hydrochloride (BAEE) and porcine trypsin were purchased from Sigma Aldrich (Louis, USA); Oligo synthesis, sequencing and modified Bradford protein assay kit were purchased from Sangong Biotech Co., Ltd. (Shanghai, China). Ultrafiltration 3-kDa cutoff filter was purchased from Millipore (Bedford, MA, USA); Benzamidine column (1 mL Φ1.6 × 2.5 cm), Gel column (HiLoad 16/60 Superdex 200 pg), and ÄKTA™ were purchased from GE Healthcare (Shanghai, China). The ion-exchange chromatography CM Sepharose FF, reversed-phase chromatography (SOURCE 30RPC) and other reagents were purchased from local businesses.

### Prediction of the point mutations

The mutations were predicted by web servers (PoPMuSiC 2.1 [28], DUET [27], NeEMO [29]). All of the predictions were used 1SGT as the template. Based on trypsin mutant tbcf (K101A), three web servers (PoPMuSiC 2.1, DUET, and NeEMO) were used to predict the positive mutation of five autolysis sites with a cutoff for increased ΔΔG value (PoPMuSiC, DUET) or Kcal/mol value over 1.4 (NeEMO). For NeEMO prediction, the pH and temperature were set at 6.0 and 30 °C. And R32A, K122A, and R153A mutation were individually introduced into tbcf (K101A), for the increased activity in the previous study [13].

### Plasmid and strain construction

The PCR product was generated from pPIC9K-tbcf (K101A) plasmid with the corresponding primers (Additional file 1: Table S1). The ligated plasmids were transformed into *E. coli* strain JM109, then selected by 50 mg L^−1^ kanamycin. Moreover, the constructed plasmids were linearized with restrictive enzyme SalI, then transformed into *P. pastoris* GS115 (His^−^) competent cell and screened by histidine autotrophic phenotype. Consequently, the recombinant yeast, with the high copy number of trypsin cassette, was screened by streaking the single colony on YPD plate with gradient geneticin (1 mg mL^−1^, 2 mg mL^−1^, 3 mg mL^−1^ and 4 mg mL^−1^). The q-PCR quantification was applied to quantify the copy number of trypsin cassette in recombinant yeast from 4 mg mL^−1^ geneticin plate [22].

The endogenous proteins were expressed with constitutive promoter pGAP. Firstly, the backbone vector was amplified from the pGAPZB plasmid by FpGAPZB and RpGAPZB primers. Then, the gene fragments were amplified from the genomic DNA of *P. pastoris* GS115 (His^−^) with the respective primers, and the *hac1s* (intron split *hac1* gene) were synthesized with the coding sequence of *hac1*. Finally, the construction of plasmids was performed by the DNA ligation kit. Moreover, the co-expressing plasmids pGAP-SSO2/SEC1 and pGAP-SSO2/UBC1 were constructed. Firstly, the backbone vector was generated from the pGAP-SSO2 plasmid with FpGAP-C and RpGAP-C. Then the expressing cassettes of *pGAP*^*m*^-*SEC1*-*tAOX1* and *pGAP*^*m*^-*UBC1*-*tAOX1* were separately amplified by primer FpGAPm and RAOX1 from plasmids pGAP^m^-SEC1 and pGAP^m^-UBC1, whose restrictive enzyme site AvrII was mutated by primers FpGAPmAvrII and RpGAPmAvrII. Finally, the backbone plasmid and expression cassettes were assembled by DNA ligation kit. With the same method, the plasmid pGAP-SEC1/UBC1 was constructed with corresponding primers. For the construction of plasmid pGAP-SSO2/SEC1/UBC1, the pGAP-SSO2/SEC1 plasmid was digested by restrictive enzyme BamHI, and the expression cassette *pGAP*^*m*^-*UBC1*-*tAOX1* was amplified from the pGAP^m^-UBC1 plasmid with primers FpGAPm-UBC1 and RpGAPm-UBC1. Finally, the digested plasmid and pGAP^m^-UBC1 expression cassette were assembled by DNA ligation kit. For constructing recombination yeast strain, the 2 ug of AvrII-linearized DNA fragment was transformed into GS115-tbcf (K101A, R201V) competent cells by electroporation. Then the transformants were incubated in YPD medium plus 1 M sorbitol (YPDS) for 2 h at 30 °C. Finally, the pellets were spread on YPDS agar plate with 80 mg L^−1^ Zeocin and grew for 3 days at 30 °C. The strains used in this study were shown in Additional file 1: Table S2.

### Media and cultivation

The media included Luria–Bertani (LB) medium, Yeast Extract Peptone Dextrose (YPD) medium, Buffered Methanol-complex (BMMY) medium and Basal Salts (BSM) medium [13]. The yeast cells were pre-cultured in YPD medium for 24 h, at 30 °C and 220 rpm. Then the pellets were resuspended in BMMY medium for 144 h cultivation with the same condition. Also, 1% methanol (v/v) was added into medium every 24 h for inducing the pAOX1 promoter. The scale-up cultivation was carried out in 3-L bio-reactor (INFORS, Switzerland), with 800 mL BSM medium. The cultivation process was divided into three phases. During the glycerol batch cultivation, the yeast cells were cultured under pH 5.5, 30 °C, and the dissolved oxygen (DO) controlled over 30% by constant agitation speed. Glycerol fed-batch cultivation was carried out when the glycerol was depleted with DO over 50%. And the feeding solution (50% glycerol with 1.2% PTM1 solution) was gradually added into medium to confer high-density cultivation with increased DO level by increased agitation speed (800 rpm). In the methanol fed-batch phase, the trypsin was expressed by methanol induced promoter pAOX1. In order to avoid the repression effect of pAOX1 by the glycerol, the inducer methanol was added 2 h later when DO was over 60%. And the methanol was gradually fed, according to the method developed by Wang et al. [46].

### Expression of human insulin precursor

The codon-optimized coding sequence of a recombinant human insulin precursor (*rPI*) was ligated into the pPICK-9 K plasmid, by the restrictive enzyme site EcoRI and BamHI. The recombinant strain GS115-rPI was screened by 4 mg mL^−1^ geneticin for the high copy number of expression cassettes. Moreover, the scale-up cultivation and preparation of insulin precursors were according to the reported method [4, 47]. The insulin precursor sample was purified by ion-exchange chromatography and reversed-phase chromatography, then lyophilized after isoelectric precipitation.

### Purification of trypsin

The culture supernatant was separated for purifying the trypsin, under centrifugation at 5000*g* for 10 min. And the filtered (0.22 μm) sample was loaded into 1 mL benzamidine column, then equilibration with buffer A (pH 7.4 50 mM Tris–HCl, 0.5 M NaCl). Finally, the trypsin was eluted with 60% buffer B (pH 2.0, 10 mM HCl, 20 mM NaAc) [13]. Moreover, the purified trypsin was further separated using the HiLoad 16/60 Superdex 200 pg column. Finally, the concentration of purified trypsin was determined by the modified Bradford protein assay kit.

### MALDI–TOF–MS analysis

MALDI-TOF–MS was applied to identify the autolysis fragments. The concentrated hydrolysate of tbcf (K101A) [13] was loaded on the MALDI-TOF–MS plate with the control without hydrolysis. Moreover, the autoproteolytic peptides were analyzed with the Swiss-Prot database [48].

### Molecular dynamics (MD) analysis

The three-dimensional model of trypsin mutant was simulated by NAMD 2.11 with CHARMM27 force field [49, 50]. For the simulation of trypsin mutants tbcf and tbcf (K101A, R201V), the modeling template SGT (PDB, 1SGT) was download from RCSB Protein Data Bank [51]. The protein was set in a cubic water box of 70 × 70 × 70 Å with the criterion that the more than 7 Å water layer in each dimension and 12 Å cut-off for non-bonded interactions. The default pH was set at 7.0 and the Na^+^ was added for charge neutralization. After water equilibration (1 ns) and minimization (1000 steps), the 60 ps of heating was performed from 0 K up to 300 K before each primary molecular simulation [52]. The temperature was kept at 300 K for 150 ps for equilibration and 10 ns simulation for data sampling at constant temperature and pressure. Finally, the three-dimensional structure was analyzed and presented by PyMOL Molecular Graphics system [53].

### Determination of trypsin activity and hydrolysis of insulin precursor

The culture supernatant or the purified enzyme were prepared to measure the trypsin activity, according to the published method [22]. And, the purified recombinant SGT and commercial porcine trypsin were prepared to hydrolyze the insulin precursor. The trypsin, with 3000 U BAEE activity, was added into the 10 mL insulin precursor solution (60 mg mL^−1^ in 50 mM pH 8.0 Tris–HCl solution with EDTA·2Na). The hydrolysis was performed at 25 °C for 19 h, then ceased by adjusting pH value to 3.0 with HCl. At the same time, the hydrolysis solution without trypsin was set as control. Consequently, the hydrolysis product was analyzed by HPLC with the modified method developed by Richard et al. [54].

All experiments were carried out with triplicate and data was shown as mean ± standard deviation.

## Supplementary information

**Additional file 1. Additional tables and figures.**

## Data Availability

All data generated or analyzed during this study are included in this published article and its additional file.

## Abbreviations

SGT: *Streptomyces griseus* trypsin
UPR: Unfolded protein response
R: Arginine
K: Lysine
BT: Bovine trypsin
MD: Molecular dynamics
ERAD: Reticulum-associated degradation
RMSD: Root-mean-square deviations
ER: Endoplasmic reticulum
DCW: Dried cell weight
DO: Dissolved oxygen
