# Supplementary material for: Improving production of Streptomyces griseus trypsin for enzymatic processing of insulin precursor
Source: Microb Cell Fact. 2020 Apr 13;19:88. doi: 10.1186/s12934-020-01338-9 (PMC7155311; doi:10.1186/s12934-020-01338-9)
Supplement: Supplementary file 1 — Additional file 1. Additional tables and figures. [file 12934_2020_1338_MOESM1_ESM.docx]

***Additional file***

**­Improving production of *Streptomyces griseus* trypsin for its application in processing insulin precursor**

Yunfeng Zhang^1, 2, 3^, Qixing Liang^1^, Chuanzhi Zhang^4^, Juan Zhang^1^, Guocheng Du^1, 2^ & Zhen Kang^1, 2^*

^1^Key Laboratory of Industrial Biotechnology, Ministry of Education, Jiangnan University, 1800 Lihu Road, Wuxi, Jiangsu 214122, China;

^2^The Key Laboratory of Carbohydrate Chemistry and Biotechnology, Ministry of Education, School of Biotechnology, Jiangnan University, Wuxi 214122, China

^3^Center for Synthetic Biochemistry, Institute of Synthetic Biology, Shenzhen Institutes of Advanced Technologies, Shenzhen, China.

^4^Bio-Pharmaceutical Research Institute Lian Yun Gang, Chia Tai Tianqing Pharmaceutical Group Co., Ltd., Lianyungang, Jiangsu, China.

^*^Correspondence and requests for materials should be addressed to Z.K. (email: zkang@jiangnan.edu.cn).

**Table S1.** Oligonucleotides used in this study

| **Primer name** | **Sequence of primer (5'→3')** |
| --- | --- |
| FR21I | ATTGCCGCCCAGGGCGAGTT |
| FR21L | TTGGCCGCCCAGGGCGAGTT |
| FR21E | GAAGCCGCCCAGGGCGAGTT |
| FR21G | GGTGCCGCCCAGGGCGAGTT |
| FR21P | CCAGCCGCCCAGGGCGAGTT |
| FR21M | ATGGCCGCCCAGGGCGAGTT |
| FR21V | GTTGCCGCCCAGGGCGAGTT |
| RR21 | GGTTCCGCCGACGACGAA |
| FR32A | GCTCTCTCCATGGGCTGCGG |
| FR32G | GGTCTCTCCATGGGCTGCGG |
| FR32S | TCTCTCTCCATGGGCTGCGG |
| FR32F | TTTCTCTCCATGGGCTGCGG |
| FR32Y | TACCTCTCCATGGGCTGCGG |
| FR32W | TGGCTCTCCATGGGCTGCGG |
| RR32 | GACCATGAAGGGGAACTCG |
| FK122A | GCTATCGCCACCACCACCGC |
| FK122I | ATTATCGCCACCACCACCGC |
| FK122L | TTGATCGCCACCACCACCGC |
| FK122E | GAAATCGCCACCACCACCGC |
| FK122V | GTTATCGCCACCACCACCGC |
| FK122Y | TACATCGCCACCACCACCGC |
| RK122 | CAGCGTGGGCTGGTTGATG |
| FR153I | ATTTACCTGCTCAAGGCCAACGTC |
| FR153L | TTGTACCTGCTCAAGGCCAACGTC |
| FR153V | GTTTACCTGCTCAAGGCCAACGTC |
| FR153P | CCATACCTGCTCAAGGCCAACGTC |
| FR153H | CATTACCTGCTCAAGGCCAACGTC |
| FR153A | GCTTACCTGCTCAAGGCCAACGTC |
| RR153 | CTGCTGGCTGCCGCCCT |
| FR201I | ATTAAGGACAACGCCGACGAGT |
| FR201L | TTGAAGGACAACGCCGACGAGT |
| FR201F | TTTAAGGACAACGCCGACGAGT |
| FR201V | GTTAAGGACAACGCCGACGAGT |
| FR201W | TGGAAGGACAACGCCGACGAGT |
| FR201E | GAAAAGGACAACGCCGACGAGT |
| FR201Y | TACAAGGACAACGCCGACGAGT |
| FR201S | TCTAAGGACAACGCCGACGAGT |
| FR201N | CAAAAGGACAACGCCGACGAGT |
| FR201G | GGTAAGGACAACGCCGACGAGT |
| FpGAPZB | GGTACCTCGAGCCGCGGC |
| RpGAPZB | CGTTTCGAAGTTGTTCAATTG |
| RR201 | GAACATCGGGCCGCCG |
| FERO1 | CTTCGAAACGATGAGGATAGTAAGGAGC |
| RERO1 | AGGTACCTTACAAGTCTACTCTATATGTGG |
| FPDI | CTTCGAAACGATGCAATTCAACTGGAATATTAAAACTGTG |
| RPDI | AGGTACCTTAAAGCTCGTCGTGAGCGTC |
| FUBC1 | CTTCGAAACGATGACTAGCATTAAACGAATC |
| RUBC1 | AGGTACCTTATCTCAAAAGCTCTTCTAAAATTTC |
| FGSH2 | CTTCGAAACGATGATGTTCCCCTTTCGTAG |
| RGSH2 | AGGTACCTTAGAAAAGATAAACGCTGTCAAC |
| FGLR1 | CTTCGAAACGATGCCCTCAATTGCCCAG |
| RGLR1 | AGGTACCTTAAACCATTGTCACTAATTCTTCAGC |
| FHSP90 | CTTCGAAACGATGTCAAAGTCCGAATCGTAC |
| RHSP90 | AGGTACCTTAGTCAACCTCCTCCATGGC |
| FBIP | CTTCGAAACGATGTTTTTCAACAGACTAAGC |
| RBIP | AGGTACCTTACAATTCGTCGTGTTCGAAATAATC |
| FSEC53 | CTTCGAAACGATGTCGTTTTCTAATAAAGAAGATC |
| RSEC53 | AGGTACCTTACAGGGAAAAGAGCTCCTTTAAG |
| FSLY1 | CTTCGAAACGATGCTTCATTTGAATGAGCCC |
| RSLY1 | AGGTACCTTATTTTGCTTCGGCACCGAG |
| FSEC1 | CTTCGAAACGATGGCTTCTGATCTGATTAAC |
| RSEC1 | AGGTACCTTATTTCCAAAATTTCTTCAGC |
| FSSO2 | CTTCGAAACGATGAGTAACCAGTATAATCCGTATG |
| RSSO2 | AGGTACCTTATCTTCCCCAGTTTCCGAC |
| FpGAPmAvrII | GTTACCGTCCTTAGGAAATTTTACTCTGC |
| RpGAPmAvrII | GGGCGGTGGAAGGAGAGA |
| FpGAPm | GTACAGAAGATTAAGTGAGAAAGAGTTGGTAGCTCTTG |
| RAOX1 | GGGATCCGCACAAACGAAGGTCTCACTTAATCTTCTGTACTC |
| FpGAP-C | CCTTCGTTTGTGCGGATC |
| RpGAP-C | TCTCACTTAATCTTCTGTACTC |
| FpGAPm-UBC1 | GTGAGACCTTCGTTTGTGCGGATCCCTCAAGAAGATCCTTTGATC |
| RpGAPm-UBC1 | TGAAGCTATGGTGTGTGGGGGATCCCAAACGAAGGTCTCACTTAATC |
| Fsgt | ACACTGGTGGCGTTGATAC |
| Rsgt | CGATGCCGACCTGAATCC |
| Fgapdh | ACAAGGACTGGAGAGGTGGTAG |
| Rgapdh | CGGTTGGGACACGGAAAGC |

**Table S2.** The Plasmids and strains used in this study

| **Plasmids or strains** | **Genotype** | **Reference** |
| --- | --- | --- |
| **Plasmids** | | |
| pPIC9K | *HIS Amp^r^ or Kan^r^* | Invitrogen |
| pPIC9K-tbcf (K101A) | pPIC9K derivative containing P*_AOX1_*-tbcf (K101A) expression cassette | *^[1]^* |
| pPIC9K-*rSGT* | pPIC9K derivative containing P_AOX1_-rSGT | *_[2]_* |
| pMD19-T-*GAPDH* | pMD19-T derivative containing the *GAPDH* gene | _[2]_ |
| pGAPZB | Expression plasmid with Zeocin selection marker | Invitrogen |
| **Strains** | | |
| *E. coli* JM109 | F´*traD36 proA*^+^*B*^+^ *lacIq Δ(lacZ) M15/Δ(lac-proAB) glnV44e14*- *gyrA96 recA1 relA1 endA1 thi hsdR17* | NEB |
| GS115(wild type) | *his4* Mut^+^ His^-^ (aox1^+^, aox2^+^) | Invitrogen |
| GS115-tbcf | GS115 *his4*∷P*_AOX1_*-*αMF(Δ57-70)-HL28-Ap-btc-EIF* *HIS, KAN* | [1] |
| GS115-tbcf (K101A) | GS115 *his4*∷P*_AOX1_*-*αMF(Δ57-70)-HL28-Ap-btc-EIF (K101A) HIS, KAN* | [1] |
| GS115-tbcf (K101A, R21P) | GS115 *his4*∷P*_AOX1_*-*αMF(Δ57-70)-HL28-Ap-btc-EIF (K101A, R21P) HIS, KAN* | This study |
| GS115-tbcf (K101A, R21M) | GS115 *his4*∷P*_AOX1_*-*αMF(Δ57-70)-HL28-Ap-btc-EIF (K101A, R21M)* *HIS, KAN* | This study |
| GS115-tbcf (K101A, R21V) | GS115 his4∷P_AOX1_-*αMF(Δ57-70)-HL28-Ap-btc-EIF (K101A, R21V)* *HIS, KAN* | This study |
| GS115-tbcf (K101A, R21S) | GS115 his4∷P_AOX1_-*αMF(Δ57-70)-HL28-Ap-btc-EIF (K101A, R21S)* *HIS, KAN* | This study |
| GS115-tbcf (K101A, R21L) | GS115 his4∷P_AOX1_-*αMF(Δ57-70)-HL28-Ap-btc-EIF (K101A, R21L)* *HIS, KAN* | This study |
| GS115-tbcf (K101A, R21E) | GS115 his4∷P_AOX1_-*αMF(Δ57-70)-HL28-Ap-btc-EIF (K101A, R21E)* *HIS, KAN* | This study |
| GS115-tbcf (K101A, R21I) | GS115 his4∷P_AOX1_-*αMF(Δ57-70)-HL28-Ap-btc-EIF (K101A, R21I) HIS, KAN* | This study |
| GS115-tbcf (K101A, R32A) | GS115 his4∷P_AOX1_-*αMF(Δ57-70)-HL28-Ap-btc-EIF (K101A, R32A)* *HIS, KAN* | This study |
| GS115-tbcf (K101A, R32F) | GS115 his4∷P_AOX1_-*αMF(Δ57-70)-HL28-Ap-btc-EIF (K101A, R32F)* *HIS, KAN* | This study |
| GS115-tbcf (K101A, R32S) | GS115 his4∷P_AOX1_-*αMF(Δ57-70)-HL28-Ap-btc-EIF (K101A, R32S)* *HIS, KAN* | This study |
| GS115-tbcf (K101A, R32Y) | GS115 his4∷P_AOX1_-*αMF(Δ57-70)-HL28-Ap-btc-EIF (K101A, R32Y)* *HIS, KAN* | This study |
| GS115-tbcf (K101A, R32W) | GS115 his4∷P_AOX1_-*αMF(Δ57-70)-HL28-Ap-btc-EIF (K101A, R32W)* *HIS, KAN* | This study |
| GS115-tbcf (K101A, R32G) | GS115 his4∷P_AOX1_-*αMF(Δ57-70)-HL28-Ap-btc-EIF (K101A, R32G) HIS, KAN* | This study |
| GS115-tbcf (K101A, K122A) | GS115 his4∷P_AOX1_-*αMF(Δ57-70)-HL28-Ap-btc-EIF (K101A, K122A)* *HIS, KAN* | This study |
| GS115-tbcf (K101A, K122L) | GS115 his4∷*P_AOX1_-αMF(Δ57-70)-HL28-Ap-btc-EIF (K101A, K122L)* *HIS, KAN* | This study |
| GS115-tbcf (K101A, K122I) | GS115 his4∷*P_AOX1_-αMF(Δ57-70)-HL28-Ap-btc-EIF (K101A, K122I) HIS, KAN* | This study |
| GS115-tbcf (K101A, K122V) | GS115 his4∷P_AOX1_-*αMF(Δ57-70)-HL28-Ap-btc-EIF (K101A, K122V)* *HIS, KAN* | This study |
| GS115-tbcf (K101A, K122Y) | GS115 his4∷P_AOX1_-αMF(*Δ*57-70)-HL28-Ap-btc-EIF (K101A, K122Y) *HIS, KAN* | This study |
| GS115-tbcf (K101A, K122E) | GS115 his4∷P_AOX1_-αMF(*Δ*57-70)-HL28-Ap-btc-EIF (K101A, K122E) *HIS, KAN* | This study |
| GS115-tbcf (K101A, R153A) | GS115 his4∷P_AOX1_-*αMF(Δ57-70)-HL28-Ap-btc-EIF (K101A, R153A) HIS, KAN* | This study |
| GS115-tbcf (K101A, R153P) | GS115 his4∷P_AOX1_-*αMF(Δ57-70)-HL28-Ap-btc-EIF (K101A, R153P)* *HIS, KAN* | This study |
| GS115-tbcf (K101A, R153I) | GS115 his4∷P_AOX1_-*αMF(Δ57-70)-HL28-Ap-btc-EIF (K101A, R153I)* *HIS, KAN* | This study |
| GS115-tbcf (K101A, R153H) | GS115 his4∷P_AOX1_-*αMF(Δ57-70)-HL28-Ap-btc-EIF (K101A, R153H)* *HIS, KAN* | This study |
| GS115-tbcf (K101A, R153V) | GS115 his4∷_PAOX1_-*αMF(Δ57-70)-HL28-Ap-btc-EIF (K101A, R153V) HIS, KAN* | This study |
| GS115-tbcf (K101A, R153L) | GS115 his4∷P_AOX1_-*αMF(Δ57-70)-HL28-Ap-btc-EIF (K101A, R153L) HIS, KAN* | This study |
| GS115-tbcf (K101A, R201V) | GS115 his4∷P_AOX1_-*αMF(Δ57-70)-HL28-Ap-btc-EIF (K101A, R201V) HIS, KAN* | This study |
| GS115-tbcf (K101A, R201I) | GS115 his4∷P_AOX1_-*αMF(Δ57-70)-HL28-Ap-btc-EIF (K101A, R201I)* *HIS, KAN* | This study |
| GS115-tbcf (K101A, R201S) | GS115 his4∷P_AOX1_-αMF*(Δ57-70)-HL28-Ap-btc-EIF (K101A, R201S) HIS, KAN* | This study |
| GS115-tbcf (K101A, R201E) | GS115 his4∷P_AOX1_-*αMF(Δ57-70)-HL28-Ap-btc-EIF (K101A, R201E) HIS, KAN* | This study |
| GS115-tbcf (K101A, R201N) | GS115 his4∷P_AOX1_-*αMF(Δ57-70)-HL28-Ap-btc-EIF (K101A, R201N)* *HIS, KAN* | This study |
| GS115-tbcf (K101A, R201G) | GS115 his4∷P_AOX1_-*αMF(Δ57-70)-HL28-Ap-btc-EIF (K101A, R201G)* *HIS, KAN* | This study |
| GS115-tbcf (K101A, R201W) | GS115 his4∷P_AOX1_*-αMF(Δ57-70)-HL28-Ap-btc-EIF (K101A, R201W)* *HIS, KAN* | This study |
| GS115-tbcf (K101A, R201F) | GS115 his4∷P_AOX1_-*αMF(Δ57-70)-HL28-Ap-btc-EIF (K101A, R201F) HIS, KAN* | This study |
| GS115-tbcf (K101A, R201L) | GS115 his4∷P_AOX1_-*αMF(Δ57-70)-HL28-Ap-btc-EIF (K101A, R201L) HIS, KAN* | This study |
| GS115-tbcf (K101A, R201Y) | GS115 his4∷P_AOX1_-*αMF(Δ57-70)-HL28-Ap-btc-EIF (K101A, R201Y) HIS, KAN* | This study |
| GS115-tbcf (K101A, R201V, R32A) | GS115 *HIS*∷*P_AOX1_-αMF(Δ57-70)-HL28-Ap-btc-EIF (K101A, R201V, R32A)* *HIS, KAN* | This study |
|  |  | This study |
|  |  |  |
|  |  |  |
| GS115-tbcf (K101A, R201V)-Bip | GS115-tbcf (K101A, R201V); pGAP-*Bip*-TAOX1 *HIS, KAN*, Zeocin | This study |
| GS115-tbcf (K101A, R201V)-Hsp90 | GS115-tbcf (K101A, R201V); pGAP-*Hsp90*-TAOX1 *HIS, KAN*, Zeocin | This study |
| GS115-tbcf (K101A, R201V)-HAC1s | GS115-tbcf (K101A, R201V); pGAP-*HAC1s*-TAOX1 *HIS, KAN*, Zeocin | This study |
| GS115-tbcf (K101A, R201V)-Ero1 | GS115-tbcf (K101A, R201V); pGAP-*Ero1*-TAOX1 *HIS, KAN*, Zeocin | This study |
| GS115-tbcf (K101A, R201V)-SLY1 | GS115-tbcf (K101A, R201V); pGAP-*SLY1*-TAOX1 *HIS, KAN*, Zeocin | This study |
| GS115-tbcf (K101A, R201V)-SEC53 | GS115-tbcf (K101A, R201V); pGAP-*SEC53*-TAOX1 *HIS, KAN*, Zeocin | This study |
| GS115-tbcf (K101A, R201V)-SEC1 | GS115-tbcf (K101A, R201V); pGAP-*SEC1*-TAOX1 *HIS, KAN*, Zeocin | This study |
| GS115-tbcf (K101A, R201V)-SSO2 | GS115-tbcf (K101A, R201V); pGAP-*SSO2*-TAOX1 *HIS, KAN*, Zeocin | This study |
| GS115-tbcf (K101A, R201V)-GLR1 | GS115-tbcf (K101A, R201V); pGAP-*GLR1*-TAOX1 *HIS, KAN*, Zeocin | This study |
| GS115-tbcf (K101A, R201V)-PDI | GS115-tbcf (K101A, R201V); pGAP-*PDI*-TAOX1 *HIS, KAN*, Zeocin | This study |
| GS115-tbcf (K101A, R201V)-GSH2 | GS115-tbcf (K101A, R201V); pGAP-*GSH2*-TAOX1 *HIS, KAN*, Zeocin | This study |
| GS115-tbcf (K101A, R201V)-UBC1 | GS115-tbcf (K101A, R201V); pGAP-*UBC1*-TAOX1 *HIS, KAN*, Zeocin | This study |
| GS115-tbcf (K101A, R201V)_SS | GS115-tbcf (K101A, R201V); pGAP-*SSO2*-TAOX1; pGAP^m^-*SEC1-*TAOX1 *HIS, KAN*, Zeocin | This study |
| GS115-tbcf (K101A, R201V)_SU | GS115-tbcf (K101A, R201V); pGAP-*SSO2*-TAOX1; pGAP^m^-*UBC1-*TAOX1 *HIS, KAN*, Zeocin | This study |
| GS115-tbcf (K101A, R201V)_US | GS115-tbcf (K101A, R201V); pGAP-*UBC1*-TAOX1; pGAP^m^-*SEC1-*TAOX1 *HIS, KAN*, Zeocin | This study |
| GS115-tbcf (K101A, R201V)_SSU | GS115-tbcf (K101A, R201V); pGAP-*SSO2*-TAOX1; pGAP^m^-*SEC1-*TAOX1; pGAP^m^-*UBC1*-TAOX1 *HIS, KAN*, Zeocin | This study |
| GS115-rPI | GS115-pAOX1-*rPI*-TAOX1 *HIS, KAN* | This study |

**Table S3.** Kinetic parameters of tbcf and tbcf (K101A, R201V) mutant

| **Enzyme** | ***K*_m_ (mM)** | ***k*_cat_ (min^-1^)** | ***k*_cat_/*K*_m_**  **（min^1^·mM^-1^）** |
| --- | --- | --- | --- |
| tbcf | 5.86 ± 0.16×10^-2^ | 7.20 ± 0.42×10^5^ | 1.23×10^7^ |
| tbcf (K101A, R201V) | 5.39 ± 0.36×10^-2^ | 8.24 ± 0.15×10^5^ | 1.53×10^7^ |


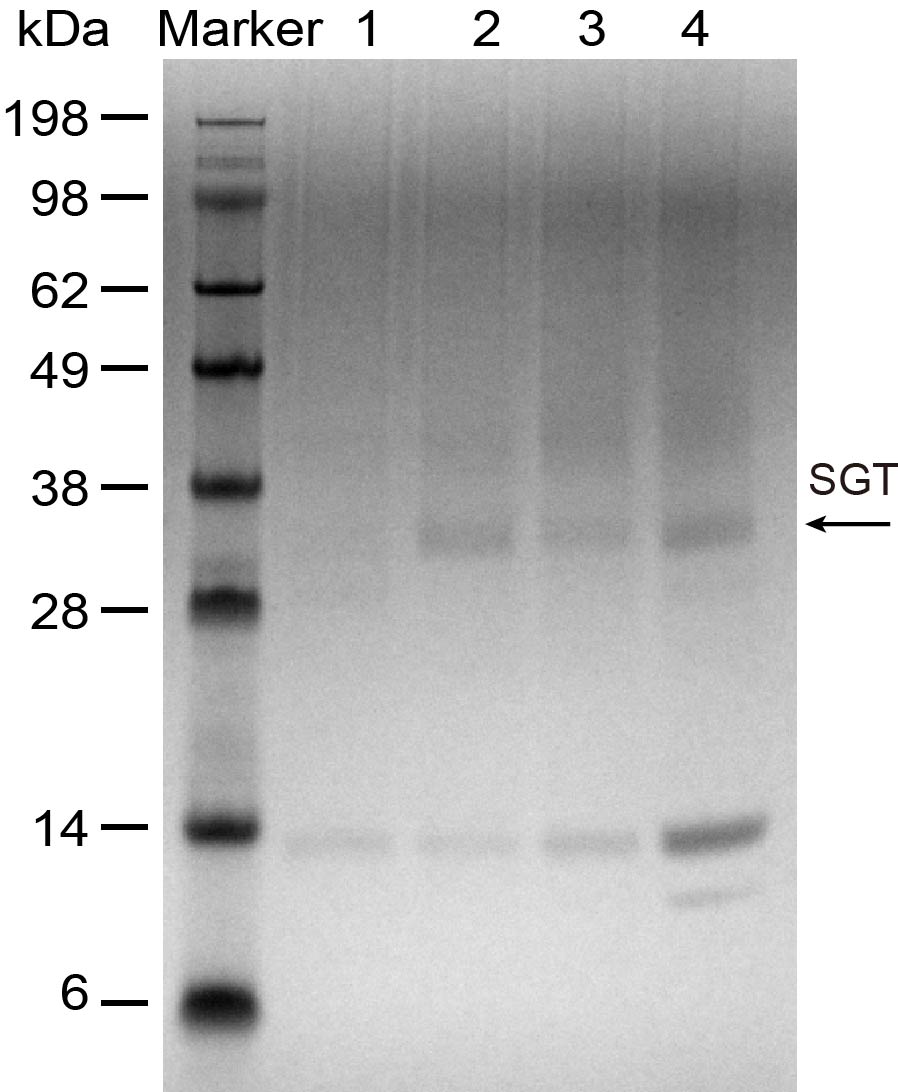


**Figure S1.** SDS-PAGE of the culture supernatant. 1, tbcf (tbcf); 2, tbcf (K101A); 3, tbcf (K101A, R201V). 4, tbcf (K101A, R201V, R32A)


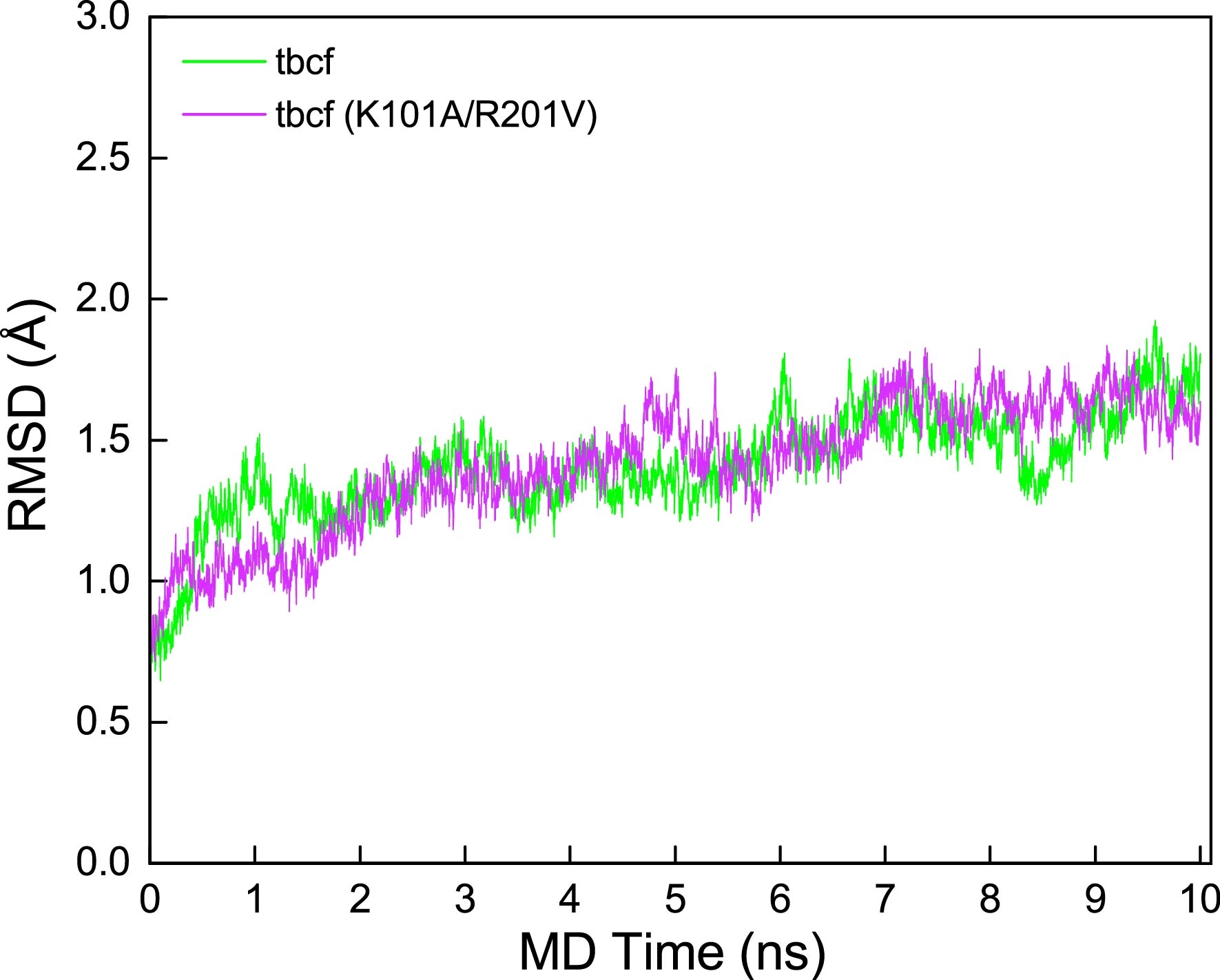


**Figure S2**. The Root-mean-square deviation (RMSD) plot by molecular dynamics simulation.

**REFERENCES**

1. Zhang Y, Huang H, Yao X, Du G, Chen J, Kang Z: High-yield secretory production of stable, active trypsin through engineering of the N-terminal peptide and self-degradation sites in *Pichia pastoris*. *Bioresour Technol* 2017, 247:81-87.

2. Zhang Y, Ling Z, Du G, Chen J, Kang Z: Improved production of active *Streptomyces griseus* trypsin with a novel auto-catalyzed strategy. *Sci Rep* 2016, 6:23158.
